# Correlation between *Helicobacter pylori* infection and severity of gastritis in children

**DOI:** 10.1128/spectrum.00312-25

**Published:** 2025-07-29

**Authors:** Wei Zheng, Xiaoyu Chen, Yaofeng Yang, Kerong Peng, Fubang Li, Hong Zhao, Weizhong Gu, Mizu Jiang

**Affiliations:** 1 Department of Gastroenterology, Children's Hospital, Zhejiang University School of Medicine, National Clinical Research Center for Child Health, National Children’s Regional Medical Center 26441 , Hangzhou, China; 2 Department of Pathology, Children's Hospital, Zhejiang University School of Medicine, National Clinical Research Center for Child Health, National Children’s Regional Medical Center 26441 , Hangzhou, China; 3 Pediatric Endoscopy Center and Gastrointestinal Laboratory, Children’s Hospital, Zhejiang University School of Medicine, National Clinical Research Center for Child Health, National Children’s Regional Medical Center 26441 , Hangzhou, China; Michigan State University, East Lansing, Michigan, USA

**Keywords:** *Helicobacter pylori*, gastritis, children, endoscopy, histopathology

## Abstract

*Helicobacter pylori* is widely recognized as the chronic infection that is most commonly observed across all age cohorts worldwide and is closely linked to chronic gastritis. In pediatric *H. pylori* patients, we aim to examine the endoscopic and histopathological alterations in the gastric mucosa. This was a potential monocentric investigation conducted from January 2018 to August 2018, collecting 120 children who underwent gastroscopy due to symptoms of gastrointestinal distress. Endoscopic procedures were conducted to acquire mucosal biopsies for the purpose of histopathological analysis. The investigation comprised a cohort of 120 patients, 64 of whom were identified as having *H. pylori* infection. *The H. pylori*-infected cohort had a greater level of inflammation, activity, and lymphoid follicles than the *H. pylori*-negative cohort. A significant link was discovered between *H. pylori* infection and both nodular lesions and active gastritis. There was a significant infection link observed between the degree of inflammation and the abundance level of *Helicobacter* in the *H. pylori*-infected cohort. There was a significant link between the infection of *H. pylori* and histopathological manifestations, including active gastritis.

**IMPORTANCE:**

*H. pylori* is a significant concern of infection in the field of public health. In children, the endoscopic observations of *H. pylori* infection exhibit variation, particularly in highly endemic countries where nodular changes in the gastric mucosa are commonly observed. Nevertheless, there is ongoing debate regarding the connection between the colonization level of *H. pylori* in the gastric mucosa and the extent of histological parameters of gastritis. We mainly explored the link between the *H. pylori* colonization density in the gastric mucosa and the severity of histological parameters related to gastritis and found that there was a significant link between the infection of *H. pylori* and histopathological manifestations, including active gastritis.

## INTRODUCTION

*Helicobacter pylori* is a significant concern of infection in the field of public health and represents the prevailing chronic infection in children and adults worldwide (1, 2). It exhibits a global reach, with a notable occurrence in developing nations (3). If left untreated during childhood, the infection caused by *H. pylori* will persist into adulthood (4). *H. pylori* is a significant danger aspect for the advancement of gastric cancer, as it is responsible for 90% of mucosa-associated lymphoid tissue lymphomas (5). The International Agency for Research on Cancer has categorized *H. pylori* as a class I cancer risk factor since 1994 (6).

Numerous epidemiological investigations have repeatedly explored that *H. pylori* epidemiology in pediatric populations exhibits dynamic and evolving patterns. *H. pylori* is highly adapted to the colonization of a unique ecological niche in the deep gastric mucus layer. Several mechanisms, including bacterial, environmental, and host factors and others, are important in *H. pylori* colonization (7, 8). Bacterial virulence factors include Cag type IV secretion system, vacA allelic genotypes linked to disease, and adhesins (9, 10). Environmental factors include smoking and dietary factors (11). Host genetic factors include single-nucleotide polymorphisms in cytokine and growth factor genes encoding proteins that have been implicated in pathogenesis and their receptors, innate immune receptors shown to be activated by *H. pylori*, enzymes involved in signal transduction cascades, glycoproteins, and DNA repair enzymes (12). Gastric inflammatory phenotypes and associated gastric functions include corpus-predominant gastritis, atrophic gastritis, hypochlorhydria, high gastrin levels, and low pepsinogen I levels and ratio of pepsinogen I to pepsinogen II. These conditions are also associated with gastric dysbiosis of microbes other than *H. pylori* (11).

The *H. pylori* infection is strongly connected to the advancement of chronic gastritis and is believed to be the primary mechanism behind the persistent gastric mucosa inflammation (13). In children, the endoscopic observations of *H. pylori* infection exhibit variation, particularly in highly endemic countries where nodular changes in the gastric mucosa are commonly observed (14, 15). Nevertheless, there is ongoing debate regarding the connection between the colonization level of *H. pylori* in the gastric mucosa and the extent of histological parameters of gastritis (16, 17).

We mainly aimed to ascertain the demographic characteristics of individuals affected by *H. pylori* infection. Additionally, we sought to find the link between the *H. pylori* colonization density in the gastric mucosa and the severity of histological parameters related to gastritis.

## MATERIALS AND METHODS

### Study cohort

The current investigation included 120 participants aged less than 16 years who presented with symptoms of gastrointestinal distress, including recurrent abdominal pain, bloating, vomiting, and dyspepsia, between January 2018 and August 2018. A group of pediatric patients consisting of 69 male and 51 female individuals was admitted to the medical facility known as the Children’s Hospital of Zhejiang University School of Medicine. The exclusion criteria of the investigation comprised individuals with a medical history of sudden onset of symptoms and who had taken bismuth-containing compounds, proton-pump inhibitors (PPI), antibiotics, H_2_ receptor antagonists, or non-steroidal anti-inflammatory drugs (NSAIDs) within the preceding 4 weeks.

### Gastric biopsies and *H. pylori* testing

Electronic gastroscopy was performed on participants at the Children’s Hospital of Zhejiang University School of Medicine. Four gastric mucosa biopsies were taken from the gastric antrum in each child. The gastric antrum biopsies were utilized for various investigative procedures, including rapid urease test, histology, culture, and research into the gastric microbiota. The endoscopic observations were properly documented. The biopsies were kept at −80°C until the DNA extraction was performed. Results obtained had to satisfy one of the following two criteria in order to be adjudged as *H. pylori* infection: (i) *H. pylori* culture being positive or (ii) both urea breath and rapid urease tests being positive (18).

### Histology

The tissue samples were immersed in a solution of 10% formalin for fixation and subsequently transferred to the anatomical pathology department for technical processing under the expertise of experienced anatomical pathologists. The histological evaluation was conducted by a couple of pathologists who were blinded to the results of other investigations. The slides underwent staining with hematoxylin-eosin and were subsequently modified with Giemsa and Alcian blue plus periodic acid-Schiff (AB-PAS), as needed. The utilization of hematoxylin-eosin facilitated the observation of cellular morphology. PAS Basic Blue staining was employed to identify foci of squamous intestinal metaplasia, while Giemsa staining was utilized to ascertain the existence of *H. pylori* at the crypt’s base and at the cell’s upper pole. The gastric inflammation was measured by means of the updated Sydney categorization, taking into account the infiltration of polymorphonuclear neutrophils (which reflects the level of activity of the inflammation) and mononuclear inflammatory cells (which indicates the extent of the inflammation). The Sydney classification comprises four separate grades, with grades 0, 1, 2, and 3 denoting none, mild, moderate, and severe, respectively (19).

### DNA extraction and determination of *Helicobacter* abundance levels

DNA MiniPrep kit (Axygen, China) was used for each biopsy sample to extract community microbial genomic DNA (20). In summary, the biopsy specimens were subjected to an overnight incubation at 56°C in ATL lysis buffer supplemented with proteinase K. Subsequently, mechanical lysis was performed for a duration of 1 minute at a velocity of 6.0 m/s, employing the FastPrep equipment (MP Biomedicals, Carlsbad, CA, USA). Following purification using spin columns, 400 µL of buffer AE was used to elute the resulting lysate. The examination of DNA quality and quantity was performed employing Nanodrop (Thermo Fisher Scientific, Massachusetts, USA). The extracted DNA underwent storage at −80°C before undergoing sequencing. Next, 16S rRNA sequencing was used to determine the abundance levels of *Helicobacter* spp.

### 16S rRNA sequencing

Amplification of the V3–V4 hypervariable regions of the 16S rRNA gene was conducted utilizing a universal primer set with a barcode (341F: CCTACGGGNGGCWGCAG, 785R: GACTACHVGGGTATCTAATCC). The template DNA was standardized to an identical concentration. PCR was executed utilizing the experimental parameters as outlined by Caporaso et al. (21). PCR was performed using TaKaRa EX Taq, a high-fidelity DNA polymerase. The PCR products underwent electrophoresis in 2% agarose gels and were subsequently purified through the employment of a QIAGEN Gel Extraction Kit (QIAGEN, Germany) and homogenized at equivalent concentrations. The steps provided by the manufacturer were followed to generate a sequencing library utilizing a TruSeqR DNA PCR-Free Sample Preparation Kit (Illumina, USA), and an index code was added. The assessment of library quality was conducted through the utilization of the Agilent Bioanalyzer 2100 system and the Qubit 2.0 Fluorometer (Thermo Scientific). Sequencing of the library was performed employing an Illumina HiSeq 2500 platform (250 bp paired-end reads) at Novogene Bioinformatics Technology Co., Ltd., located in Beijing, China.

### Statistical analysis

IBM SPSS, version 20.0 (Hangzhou, China), a widely used software for data analysis, was used to conduct the statistical analyses. The count data were represented as ratios or proportions, while the measurement data were represented as means with their corresponding standard deviations (mean ± SD). The *t*-test, χ^2^ test, or Kruskal-Wallis test was utilized to conduct comparisons between the two groups. Barcodes and forward and reverse primer sequences were removed, and original sequences were examined, employing the Quantitative Insight into Microbial Ecology (QIIME), version 1.9 (22). Quality control of raw data was based on the low mass score distribution characteristics of MiSeq sequencing data. The UCHIME algorithm was employed to eliminate chimera sequences, resulting in the production of refined markers that were subsequently utilized for additional investigation (23). Version 7.0.1001 of the UPARSE pipeline was employed to conduct sequence analysis (24), assuming 97% similarity and clustering sequences into operational taxonomic units (OTU). The representative sequences of every OTU underwent a screening process for subsequent annotation. The RDP Classifier, version 2.2, was utilized to annotate the taxonomic information for each representative sequence, with reference to the Greengenes 97% data set (25). Normalization of OTU abundance data was conducted by employing a standard sequence count that corresponded to the sample with the lowest number of sequences. The normalized data obtained from QIIME was utilized to conduct subsequent diversity analyses. All *P* values were evaluated by a two-tailed test, and a significance level of *P* < 0.05 was carried out to identify statistical significance.

## RESULTS

### Characteristics of the investigation population’s demographics

This investigation examined a total of 120 patients, with 69 identified as male and 51 as female. Table 1 lays out the baseline characteristics of the patients. In our study, it was observed that out of the total of 120 patients, 64 individuals (53.33%) displayed *H. pylori* infection. The average age was 9.50 ± 3.05 years, which was comparable to the average age of the remaining 56 individuals (46.67%) who did not exhibit *H. pylori* infection (10.11 ± 3.13 years). The statistical analysis indicated that there was no significant variation in age between the two cohorts (*P* = 0.29). Among the *H. pylori*-infected cohort (*H. pylori*-associated gastritis group), there were 37 individuals who identified as male and 27 as female. Among the *H. pylori*-negative cohort (*H. pylori*-negative gastritis group), there were 32 individuals who identified as male and 24 individuals who identified as female. However, there is an absence of significant differences between the two cohorts in gender (*P* = 0.94). The cohort was stratified based on age categories, namely, early risk (0–6 years), middle risk (7–12 years), and adolescence (13–17 years). The rates of *H. pylori* infection detection were 65.22%, 50.70%, and 50.00%, respectively. The incidence of *H. pylori* infection did not increase with age and was not significant between the different age groups (*P* = 0.44).

**TABLE 1:** Comparison of demographic and clinical characteristics between the *Helicobacter pylori*-positive group and the *H. pylori*-negative group^*a*^^*,*^^*b*^

Characteristics	*H. pylori*+, *n* (%)	*H. pylori*−, *n* (%)	*t*/χ^2^	*P* value
Maternal age (years) (mean ± SD)	9.50 ± 3.05	10.11 ± 3.13	−1.074	0.29
Gender			0.005	0.94
Male	37 (57.81)	32 (57.14)	–^*c*^	–
Female	27 (42.19)	24 (42.86)	–	–
Age (years)			1.618	0.44
0–6	15 (23.44)	8 (14.29)	–	–
7–12	36 (56.25)	35 (62.50)	–	–
13–17	13 (20.31)	13 (23.21)	–	–

^*a*^ The comparison of data between the two groups was done by unpaired *t*-test or χ^2^ test.

^*b*^ *H. pylori*+, *Helicobacter pylori*-positive group; *H. pylori*−, *Helicobacter pylori*-negative group.

^*c*^ “–,” not applicable.

### Clinical characteristics and endoscopic patterns

The primary indication for endoscopy was abdominal pain; nevertheless, no significant variation was discovered between the cohorts that tested positive and negative for *H. pylori* (*P* > 0.05). Other indications for endoscopy are not significant. All indications for endoscopy are listed in Table 2. Some children have more than one indication for endoscopy.

**TABLE 2:** Reasons for endoscopy between the two groups^*a*^^*,*^^*b*^

Symptoms	*H. pylori*+ (*n*)	*H. pylori*− (*n*)	χ^2^	*P* value
Abdominal pain	53	44	0.347	0.56
Emesis	3	8	−^*c*^	0.11
Anemia	1	1	−	0.99
Halitosis	3	1	−	0.62
Lack of appetite	2	1	−	0.99
Chest congestion	1	0	−	0.99
Diarrhea	1	1	−	0.99

^*a*^ The comparison of data between the two groups was done by χ^2^ test or Fisher’s exact test.

^*b*^ *H. pylori*+, *Helicobacter pylori*-positive group; *H. pylori*−, *Helicobacter pylori*-negative group.

^*c*^ “–” indicates that chi-square values are not available.

**Fig 1:**
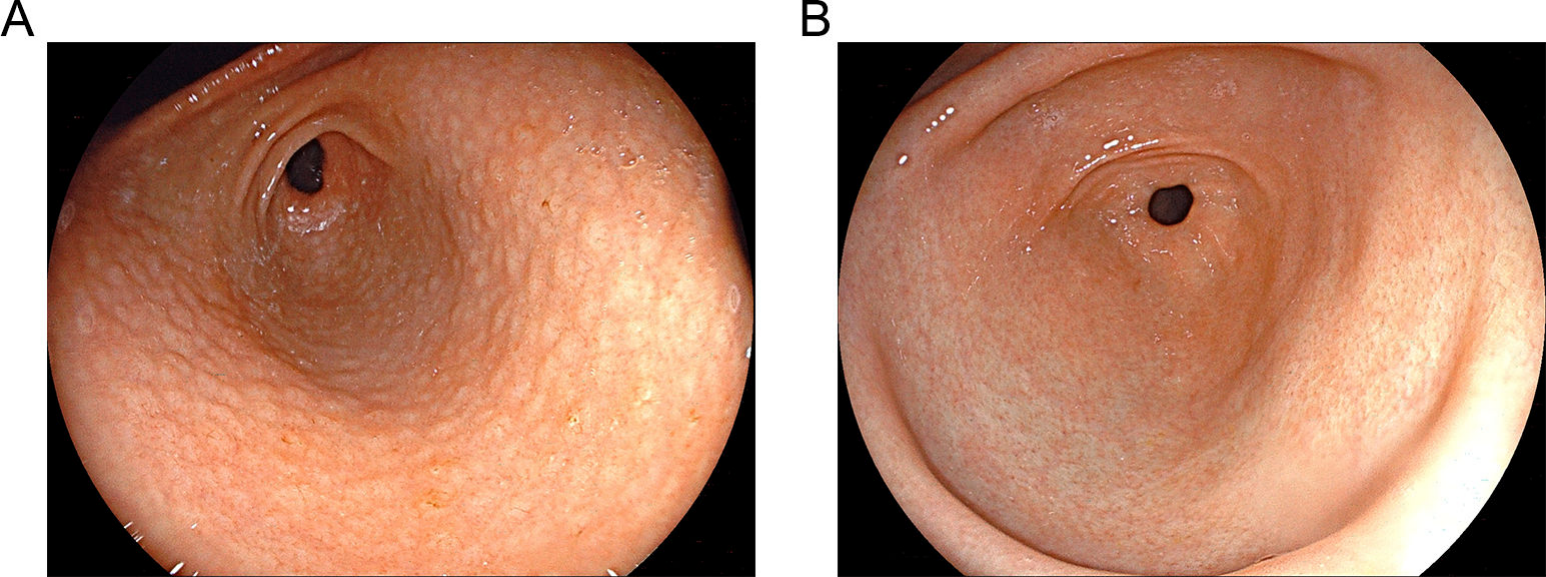
Endoscopic findings (white light). (**A**) *Helicobacter pylori*-positive group; (**B**) *H. pylori*-negative group.

The endoscopic evaluation demonstrated significant mucosal nodularity, along with hyperemia and edema, in the *H. pylori*-infected cohort, while the normal cohort showed minimal changes, hyperemia, and edema (Table 3; Fig. 1). In the *H. pylori*-infected cohort, the prevalence of gastric mucosal nodularity was found to be 40.63%, whereas in the normal group, it was only 1.79%. The observed result demonstrated statistical significance, as indicated by a *P* value of less than 0.0001. Endoscopic observation of nodular lesions was linked with the existence of *H. pylori* (*r* = 0.514, *P* < 0.0001). Only three children developed gastric ulcers, and two of them were infected with *H. pylori*.

**TABLE 3:** Endoscopic pattern of the gastric mucosa between the two groups^*a*^^*,*^^*b*^

Endoscopic pattern	*H. pylori*+ (*n*)	*H. pylori*− (*n*)	χ^2^	*P* value
Hyperemia and edema	42	30	1.808	0.18
Ulcer	2	1	−^*c*^	0.99
Erosions	2	2	−	0.99
Nodularity	26	1	−	<0.0001
Minimal changes	1	22	−	<0.0001

^*a*^ The comparison of data between the two groups was done by χ^2^ test or Fisher’s exact test.

^*b*^ *H. pylori*+, *Helicobacter pylori*-positive group; *H. pylori*−, *Helicobacter pylori*-negative group.

^*c*^ “–” indicates that chi-square values are not available.

**Fig 2:**
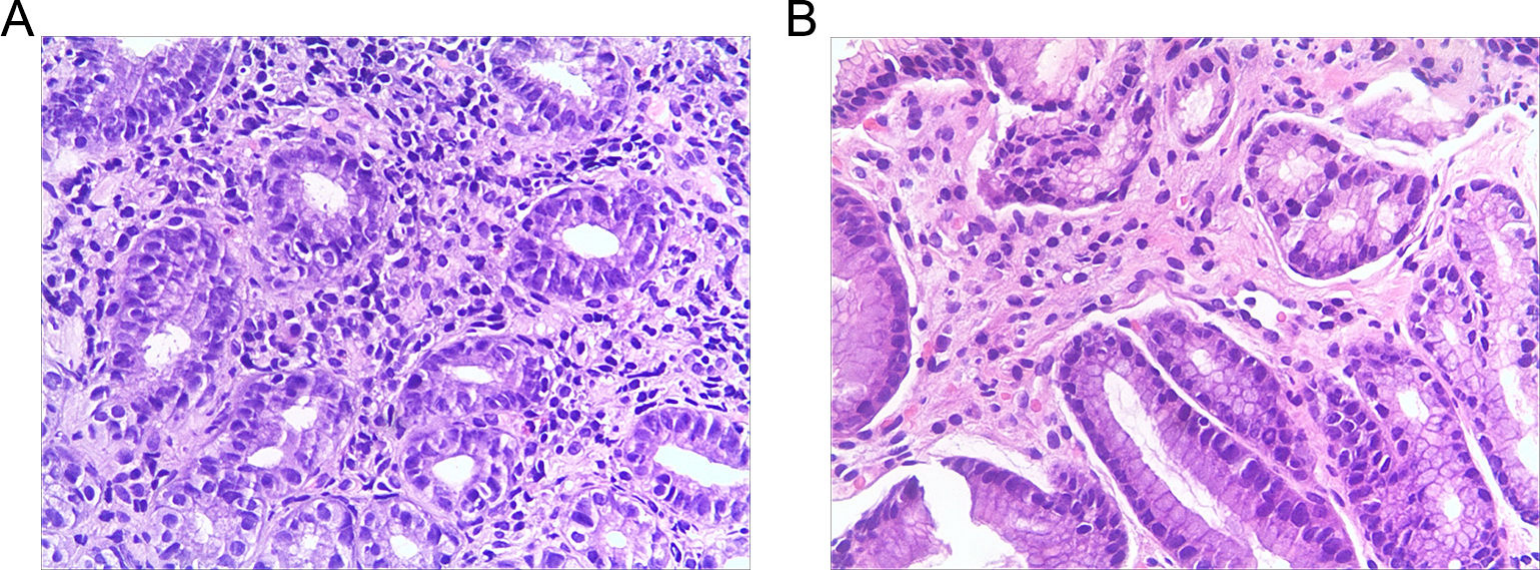
Histological findings (hematoxylin-eosin ×200). (**A**) *H. pylori*-positive group; (**B**) *H. pylori*-negative group. Gastric antral mucosa with massive chronic inflammatory cells in the *H. pylori*-positive group.

Histopathology showed that the *H. pylori*-infected cohort had a greater level of inflammation, activity, and lymphoid follicles than the *H. pylori*-negative cohort (Table 4; Fig. 2). According to the latest Sydney classification of gastritis, 89.06% of children with infection caused by *H. pylori* had chronic gastritis of moderate to severe intensity, while 85.71% of children without infection had mild to mild chronic gastritis. The incidence of chronic gastritis classified as moderate and severe was discovered to be greater in the *H. pylori*-infected cohort than in the *H. pylori*-negative group (*P* < 0.05). Glandular atrophy and intestinal metaplasia were not present in this pediatric cohort.

**TABLE 4:** Histopathological findings in the gastric mucosa between the two groups^*a*^^*,*^^*b*^

Histopathological findings	*H. pylori*+	*H. pylori*−	*t*/χ^2^	*P* value
Level of inflammation (mean ± SD)	1.98 ± 0.45	1.14 ± 0.35	11.215	<0.001
Level of activity (mean ± SD)	0.95 ± 0.42	0.31 ± 0.11	12.471	<0.001
Lymphatic follicles (mean ± SD)	0.76 ± 0.41	0.40 ± 0.24	3.695	<0.001
Positive histopathological examination for *H. pylori* (*n*)	54	0	/^*c*^	<0.001
Chronic inflammation			67.67	<0.001
Mild (*n*)	7	48	/	/
Moderate (*n*)	51	8	/	/
Severe (*n*)	6	0	/	/
Glandular atrophy (*n*)	0	0	/	/
Metaplasia (*n*)	0	0	/	/

^*a*^ The comparison of data between the two groups was done by unpaired *t*-test or Fisher’s exact test or χ^2^ test.

^*b*^ *H. pylori*+, *Helicobacter pylori*-positive group; *H. pylori*−, *Helicobacter pylori*-negative group.

^*c*^ “/,” not applicable.

### Comparison among individuals in the *H. pylori*-infected group

There was a notable infection link (*r* = 0.671, *P* < 0.0001) between the degree of inflammation and the abundance level of *Helicobacter* in the *H. pylori*-infected cohort. In the *H. pylori*-infected cohort, a total of 50 children were diagnosed with gastritis, while the remaining 14 children were diagnosed with peptic ulcers (12 of them had duodenal ulcers, and the other 2 had gastric ulcers). Abundance levels of *Helicobacter* in gastric mucosa were found to be significantly elevated in children diagnosed with gastritis compared to those diagnosed with ulcers (*t* = 9.378, *P* = 0.01; Table 5).

**TABLE 5:** Histopathological findings and *Helicobacter* abundance (16S rRNA gene sequencing) in the *Helicobacter pylori*-positive group^*a*^

Items	Ulcer group	Gastritis group	χ^2^/*t*	*P* value
Chronic inflammation (*n*)			2.052	0.36
Mild	3	4	–^*b*^	–
Moderate	10	41	–	–
Severe	1	5	–	–
*Helicobacter* abundance (mean ± SD, %)	23.23 ± 19.20	32.46 ± 22.60	9.378	0.01

^*a*^ The comparison of data between the two groups was done by χ^2^ test or unpaired *t*-test.

^*b*^ “–,” not applicable.

## DISCUSSION

*H. pylori* is widely acknowledged as a significant etiological element in the occurrence of chronic gastritis, and it has the potential to induce the formation of precancerous lesions and ultimately contribute to the advancement of gastric cancer. It is a gram-negative bacterium whose colonization starts early in life and is now considered to be the most common infectious agent in all age groups. It usually is transmitted from person to person through saliva during childhood (26–28).

Our aim was to assess the link between infection with *H. pylori* and endoscopic patterns as well as histopathological characteristics in pediatric patients. In the study, a proportion of 53.33% of the patients were included and were found to have *H. pylori* infection. Furthermore, statistical analysis revealed the absence of a significant difference in mean age between the *H. pylori*-infected group and the *H. pylori*-negative cohort (*P* = 0.29). Based on the established guidelines, it has been observed that infection of the gastric mucosa by *H. pylori* typically manifests around the age of 10 years, with a significant number of patients experiencing a prolonged period of asymptomatic infection (29). According to an investigation carried out by Rosu et al. (26), a positive link was observed between the age of the patients and the frequency of infection. Previous meta-analytic studies have yielded findings indicating a lack of a significant link between gender and the frequency of *H. pylori* infection, and our results were consistent with this literature report (30). Prior research has indicated that advanced age is correlated with a heightened vulnerability to infection (31, 32). However, in our study, there was no significant change in the rate of infection with increasing age for reasons considered to be related to ethnicity, dietary habits, sample size, and so on. Genetic variation in *H. pylori* strains can also be impactful on patient outcomes, and numerous correlative and mechanistic studies have characterized genetic elements encoded by *H. pylori* that can severely impact host pathology (33, 34).

Rosu et al. did not identify any significant positive connection between gastrointestinal symptoms, pain location, and clinical characteristics and *H. pylori* infection in children (26). Spee et al., in a meta-analysis, reported that there was no connection between clinical characteristics and infection except for gastroparesis (35). The findings of the investigation validated the absence of a significant difference in the manifestation of various discomfort symptoms between *H. pylori-*infected and *H. pylori*-negative cohorts (*P* > 0.05), which was similar to previous relevant studies based on children (31), suggesting that *H. pylori* infection has no specific clinical manifestations in childhood.

Nodular changes in the antral mucosa have been suggested in previous literature as a predictor of *H. pylori* infection in children (31, 36). Also, our findings confirmed that nodular lesions of the gastric antral mucosa were significantly connected with *H. pylori* infection (*r* = 0.514, *P* < 0.0001). Histopathology showed that the *H. pylori*-infected cohort had a larger level of inflammation than the *H. pylori*-negative group. In contrast, the low incidence of gastric ulcers associated with the infection indicates the specificity of the inflammatory response caused by *H. pylori* infection (37) and the lack of a necessary link between gastric ulcers and *H. pylori* infection (32).

A prior investigation has established a notable connection between the inflammation degree and the *H. pylori* colonization level (38). Nevertheless, the investigation carried out by Park et al. (39) did not yield any evidence of such a relationship. Our results confirmed that the severity of inflammation was significantly linked with the level of abundance of *Helicobacter* spp. Study variations were considered to be related to genetic differences between study populations, nutritional habits, and environmental factors, among others.

It is worth noting that this study had limitations. First, it is important to note that the gastrointestinal microbiota is subject to variability and can be impacted by various external factors, including medication and dietary choices. To prevent these potential confounding factors, we excluded individuals who had recently taken antibiotics, PPI, or NSAIDs. In addition, all biopsies were conducted on an empty stomach during endoscopy to reduce the possible impact of dietary intake. Second, it should be noted that the cases under investigation were exclusively obtained from the Children’s Hospital of Zhejiang University School of Medicine. Furthermore, it is important to acknowledge that the study’s sample size was restricted. Moreover, in all single-center studies, there was the possibility of unavoidable confounding variables in the results. Therefore, multicenter studies are needed to validate our results.

The findings of our study indicate a significant link between the colonization of *H. pylori* and the presence of chronicity, activity, and nodular lesions in gastritis. *H. pylori* has been found to have a significant impact on the progression and persistence of chronic active inflammation in the gastric mucosa.

## Data Availability

The data sets presented in this study can be found at https://www.ncbi.nlm.nih.gov/sra/PRJNA680429.

## SUPPLEMENTAL MATERIAL

Supplemental material spectrum.00312-25-s0001.doc Additional experimental details and Table S1.

ASM does not own the copyrights to Supplemental Material that may be linked to, or accessed through, an article. The authors have granted ASM a non-exclusive, world-wide license to publish the Supplemental Material files. Please contact the corresponding author directly for reuse.
