# Supplementary material for: Correlation between Helicobacter pylori infection and severity of gastritis in children
Source: Microbiol Spectr. 2025 Jul 29;13(9):e00312-25. doi: 10.1128/spectrum.00312-25 (PMC12403714; doi:10.1128/spectrum.00312-25)
Supplement: Supplemental material — Additional experimental details and Table S1. [file spectrum.00312-25-s0001.doc]

**Supplementary Material**

1. **Rapid urease test**

Rapid urease test is an indirect test that detects the presence of *Helicobacter. pylori* (*H. pylori*) based on the detection of urease in, or on the gastric mucosa. The biopsy specimen from the gastric antrum is placed in urea impregnated dry membrane, and in contact with the method to detect the products of urea hydrolysis, ammonia. If the reagent color changes to red within the next 10 minutes, the biopsy was deemed to be urease test positive.

1. ***H. pylori* culture**

2.1 Bacterial inoculation

Take the gastric mucosa tissue homogenate 100μl bacterial solution drops on the blood plate of heart and brain dip, L-shaped slide spread.

2.2 Bacterial culture

After the coated bacterial solution completely penetrated into the culture medium, the plate was placed in an anaerobic incubator at 37℃ for 3~7 days under microaerobic conditions (5% O_2_, 10% CO_2_, 85% N_2_).

2.3 Smear staining

Take out the *H. pylori* petri dish from the anaerobic box, observe the morphology of the colonies on the plate, the colonies were seen to be translucent with the size of the tip of a needle, select the colonies to smear, fix with alcohol lamp over fire, Gram staining, and dry the slides at 37℃ in the constant temperature box.

2.4 Microscopic identification

Observe the bacterial morphology under microscope (10x100), gram-negative Campylobacter is visible.

2.5 Fast urease test

Pick *H. pylori* positive colonies, add urease reagent, observe at room temperature for 5min, the reagent can be seen immediately from yellow to red, that is, the rapid urease test is positive.

**Table S1**. Abundance levels of *Helicobacter* spp. per child in the *Helicobacter pylori* positive groups

| Serial number Diagnosis *Helicobacter* abundance (%) |
| --- |

1 gastritis 37.00%

2 gastritis 57.01%

3 gastritis 6.55%

4 gastritis 63.22%

5 gastritis 80.99%

6  gastritis 68.00%

7 gastritis 62.53%

8 gastritis 13.48%

9 duodenal bulb ulcer 45.32%

10 duodenal bulb ulcer 12.03%

11 gastritis 0.22%

12 gastritis 34.71%

13 gastritis 0.44%

14 gastritis 29.68%

15 gastritis 51.03%

16 gastritis 47.54%

17 gastritis 28.66%

18 duodenal bulb ulcer 2.30%

19 gastritis 64.44%

20 gastritis 6.82%

21 gastritis 38.10%

22 duodenal bulb ulcer 42.09%

23 duodenal bulb ulcer 49.66%

24 gastritis 67.15%

25 gastritis 22.61%

26 gastritis 50.10%

27 gastritis 0.36%

28 gastritis 24.49%

29 gastritis 68.80%

30 duodenal bulb ulcer 51.98%

31 gastritis 0.64%

32 gastritis 33.83%

33 gastritis 12.28%

34 gastritis 15.11%

35 gastritis 47.46%

36 gastritis 43.83%

37 gastritis 14.41%

38 gastritis 21.73%

39 gastritis 22.39%

40 gastritis 61.38%

41 gastritis 67.53%

42 gastritis 33.45%

43 gastritis 23.94%

44 gastritis 27.15%

45 duodenal bulb ulcer 33.68%

46 gastritis 55.03%

47 gastritic ulcer 0.46%

48 gastritis 33.84%

49 gastritis 0.63%

50 gastritis 47.46%

51 gastritis 5.65%

52 gastritis 18.73%

53 gastritis 28.86%

54 duodenal bulb ulcer 0.96%

55 gastritis 23.54%

56 duodenal bulb ulcer 0.41%

57 gastritis ulcer 26.86%

58 duodenal bulb ulcer 21.95%

59 gastritis 29.54%

60 duodenal bulb ulcer 25.87%

61 duodenal bulb ulcer 11.57%

62 gastritis 1.03%

63 gastritis 28.05%

64 gastritis 1.60%

|  |
| --- |
